# Empowering With PrEP (E-PrEP), a Peer-Led Social Media–Based Intervention to Facilitate HIV Preexposure Prophylaxis Adoption Among Young Black and Latinx Gay and Bisexual Men: Protocol for a Cluster Randomized Controlled Trial

**DOI:** 10.2196/11375

**Published:** 2018-08-28

**Authors:** Viraj V Patel, Zoë Ginsburg, Sarit A Golub, Keith J Horvath, Nataly Rios, Kenneth H Mayer, Ryung S Kim, Julia H Arnsten

**Affiliations:** ^1^ Division of General Internal Medicine Department of Medicine Montefiore Health System / Albert Einstein College of Medicine Bronx, NY United States; ^2^ Department of Family Medicine Swedish Cherry Hill Campus Swedish Medical Center Seattle, WA United States; ^3^ Hunter HIV/AIDS Research Team Department of Psychology Hunter College, City University of New York New York, NY United States; ^4^ Department of Epidemiology and Community Health School of Public Health University of Minnesota Minneapolis, MN United States; ^5^ The Fenway Institute Boston, MA United States; ^6^ Infectious Disease Fellowship Beth Israel Deaconess Medical Center Harvard Medical School Boston, MA United States

**Keywords:** pre-exposure prophylaxis, HIV, social media interventions, HIV prevention, social network intervention, social media, social networking, telemedicine

## Abstract

**Background:**

Young black and Latinx, gay, bisexual, and other men who have sex with men (YBLGBM, aged 18-29 years) have among the highest rates of new HIV infections in the United States and are not consistently reached by existing prevention interventions. Preexposure prophylaxis (PrEP), an oral antiretroviral regimen taken daily by HIV-uninfected individuals to prevent HIV acquisition, is highly efficacious in reducing HIV acquisition and could help stop the HIV epidemic in YBLGBM. Use of social media (eg, Facebook, Twitter, online dating sites) is ubiquitous among young people, providing an efficient avenue to engage YBLGBM to facilitate PrEP adoption.

**Objective:**

Our overall goal was to develop and pilot test a theoretically grounded, social media–based, peer-led intervention to increase PrEP uptake in YBLGBM. We used diffusion of innovation and information-motivation-behavioral skills frameworks to (1) identify potential factors associated with interest in and adoption of PrEP among YBLGBM; (2) develop Empowering with PrEP (E-PrEP), a social media–based, peer-led intervention to increase PrEP uptake in YBLGBM; and (3) pilot test the feasibility and acceptability of E-PrEP, and determine its preliminary efficacy for increasing adoption of PrEP by YBLGBM. We describe the development and protocol for E-PrEP.

**Methods:**

Using a participatory research approach, we partnered with YBLGBM intervention development partners to develop a social media–based behavioral intervention to facilitate PrEP uptake, which involved an online messaging campaign disseminated by YBLGBM peer leaders to their existing online networks. We designed the 6-week campaign to provide education about PrEP, increase motivation to use PrEP, and facilitate access to PrEP. We then conducted a cluster-randomized trial of E-PrEP compared with an attention-matched general health control condition (E-Health) among YBLGBM aged 18 to 29 years to assess E-PrEP’s feasibility, acceptability, preliminary efficacy for increasing self-reported intention to use PrEP, PrEP uptake, and impact on knowledge and attitudes about PrEP at 12-week follow-up (6 weeks after the end of the online campaign).

**Results:**

From October 2016 to March 2017, we developed, pretested, and refined E-PrEP with 6 YBLGBM intervention development partners. From May to June 2017, we recruited, enrolled, and randomly assigned 10 peer leaders (n=5 for each condition). The 10 peer leaders then recruited and enrolled 152 participants from their existing online networks (range 3-33 per peer leader), during June and July 2017. Intervention follow-up was completed after 12 weeks, in November 2017, with analyses underway.

**Conclusions:**

We hypothesize that, compared with E-Health, participants randomly assigned to E-PrEP will be more likely to express intention to use PrEP and greater PrEP uptake, and will also show changes in potential mediators of PrEP uptake (knowledge, attitudes, stigma, and access). A Web-based biobehavioral intervention model such as E-PrEP could be rapidly scaled even with limited resources and have significant population-level impact.

**Trial Registration:**

ClinicalTrials.gov NCT03213366; https://clinicaltrials.gov/ct2/show/NCT03213366 (Archived by WebCite at http://www.webcitation.org/71onSdcXY)

**Registered Report Identifier:**

RR1-10.2196/11375

## Introduction

### The Role of Social Media in HIV Prevention

Gay, bisexual, and other men who have sex with men (GBM) make up 2% to 3% of the adult population [1] and continue to account for the majority of the 40,000 new HIV infections occurring annually in the United States. HIV disparities affecting young GBM, and particularly young black and Latinx (a gender-neutral term sometimes used in lieu of Latino or Latina), gay, bisexual, and other men who have sex with men (YBLGBM), are even more pronounced. YBLGBM have some of the highest rates and incidences of HIV [2-5]. While many effective behavioral HIV prevention interventions have been developed, these programs often do not reach an estimated three-quarters of young GBM [6]. This lack of reach may be partly explained by the inability to engage YBLGBM who do not identify as gay or bisexual, or who are unlikely to present in person to lesbian, bisexual, gay, and transsexual– or HIV-affiliated settings or sexually transmitted infection clinics, where most interventions have traditionally taken place^.^ To reduce the burden of HIV in YBLGBM, rapid development and implementation of new prevention strategies with a broader reach are urgently needed [7-10].

Preexposure prophylaxis (PrEP) with oral antiretroviral medication is a highly effective biomedical HIV prevention strategy. In clinical trials, daily PrEP has been found to be extremely efficacious (>95% when taken daily) in preventing HIV infection in men who have sex with men, heterosexuals, and injection drug users [11-14]. Less is known about the real-world impact of PrEP on YBLGBM. Current data indicate that young black and Latinx men have lower rates of PrEP uptake than other groups of men [15], suggesting disparities in knowledge, interest, or access to this new prevention tool. Ensuring access to PrEP by YBLGBM is paramount [16,17], including facilitating access to information and resources to support decision making about PrEP use [17-20]. New scalable interventions that can rapidly disseminate information and support PrEP uptake are needed to achieve this goal and to reduce the burden of HIV in this population.

Social media may be one such tool that could help support PrEP adoption by YBLGBM. As a tool for behavioral interventions, social media employs internet-based technologies (eg, Facebook, Instagram, and Twitter) to support interactive dialogue through the exchange of user-generated content in online networks [21]. Social media access and use by young people is ubiquitous, and disparities in use by race/ethnicity or income are minimal among youth [22-24]. A prior study in low-income YBLGBM in New York , NY, USA, showed universal access to and daily use of multiple social media sites, even among homeless YBLGBM [25]. Other studies showed that YBLGBM are readily identifiable and accessible through social media, and that many use these sites to seek sex partners [26,27]. Given their high risk of acquiring HIV [24,28-32], their extensive use of mobile phones and the internet, and the failure of traditional HIV interventions to reach YBLGBM, social media may be particularly efficient for engaging this population [33].

### Objective

Although several studies of social media–based health interventions have been published [8,29,30,34-37], best practices in this field for HIV prevention are unknown and evolving. The overall goal of this study was to develop a culturally tailored, peer-led, social media–based behavioral intervention to support PrEP uptake in YBLGBM. Our aims were to use a diffusion of innovation (DOI) framework to (1) determine potential factors associated with interest in and adoption of PrEP among YBLGBM; (2) develop Empowering with PrEP (E-PrEP), a social media–based, peer-led intervention to increase PrEP uptake in YBLGBM aged 18 to 29 years in New York City; and (3) pilot test feasibility and acceptability, and determine preliminary efficacy of E-PrEP for increasing adoption of PrEP by YBLGBM. This paper describes the development of the E-PrEP intervention and the study protocol.

## Methods

### Overview

We first developed, pretested, and refined the Web-based intervention (E-PrEP) (intervention development phase). We then conducted a 2-arm cluster-randomized controlled trial to evaluate the feasibility, acceptability, and preliminary efficacy of E-PrEP, compared with an attention-matched general health control condition (E-Health). We randomly assigned YBLGBM peer leaders to either the E-PrEP intervention or the E-Health control condition. Peer leaders were trained to deliver the intervention or control condition in their assigned arm, and then recruited YBLGBM individuals from their existing online networks (network participants) to complete a Web-based screening and baseline survey. Eligible network participants were enrolled into the trial and assigned to either a private Facebook group (Facebook, Inc, Menlo Park, CA, USA) or Instagram (Instagram Inc, Menlo Park, CA, USA) feed, connected to the peer leader who recruited them. Network participants were thus assigned to the intervention or control condition based on their peer leader’s assignment. Peer leaders then launched an online campaign by posting intervention or control condition content (eg, articles, video clips, and infographics) almost daily to their respective private groups, and by attempting to engage their network participants in discussions of the materials being posted. The online campaign occurred over a 6-week period, after which participants completed an immediate postintervention assessment and another postintervention assessment after 6 additional weeks (12 weeks after the start of the campaign).

### Theoretical Models

**Figure 1:**
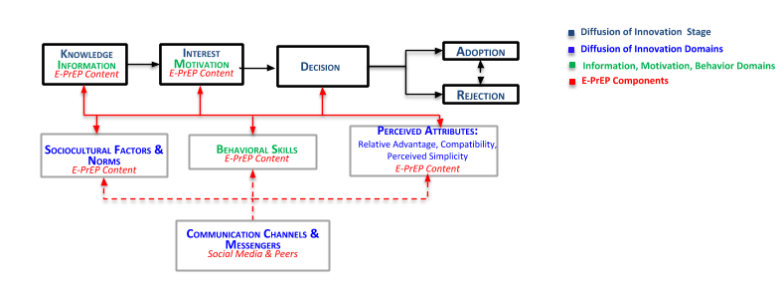
Empowering with PrEP (E-PrEP) conceptual model. DOI: diffusion of innovation; IMP: information, motivation, behavioral skills; PrEP: preexposure prophylaxis. Adapted from Fisher and Fisher [42] and Rogers [43].

We developed E-PrEP based on DOI [38] and information-motivation-behavioral skills (IMB) [39] models. The DOI model posits that a new innovation (eg, PrEP) is adopted over time through communication among members of a similar social system in a staged process, involving changes in norms and perceived attributes about the innovation [40,41]. Stages in the process include acquiring knowledge of the innovation, which may lead to interest and then a decision to adopt or reject the innovation, followed by actual adoption or rejection. In Figure 1 [42,43], we highlight elements of the innovation (PrEP) that may influence adoption, based on DOI theory. These are relative advantage (the benefits of using PrEP relative to other HIV prevention strategies), compatibility (PrEP fitting into potential users’ routines or existing behaviors), perceived simplicity (PrEP being relatively easy to acquire and use), and trialability (ability to try PrEP without long-term commitment).

While the DOI framework is highly informative, it does not explicitly provide a pathway to develop skills for adoption of an innovation (eg, navigating health care systems to obtain PrEP). Therefore, we also incorporated all components of the IMB framework [44]. The IMB model posits that fostering information acquisition, increasing motivation, and building behavioral skills are needed to change HIV prevention behaviors (eg, PrEP use) [45] and has been recently proposed as a model for guiding PrEP uptake interventions [46].

Using these 2 models as guides, we selected relevant behavioral targets to increase PrEP adoption focusing on (1) communication channels and messengers, (2) sociocultural factors and norms, and (3) perceived attributes (Figure 1). We then used a community-based participatory research approach [47-49] to develop E-PrEP messages targeting the DOI and IMB domains, and stages of knowledge and information, interest and motivation, acquisition of behavioral skills, and decision (to adopt PrEP).

### Setting

All aspects of the study took place in New York City. The HIV epidemic in New York City mirrors US national trends, with new infections disproportionately occurring among YBLGBM [50]. PrEP, which is covered by Medicaid and most other insurers in New York State, is now widely accessible in New York City. New York State has a PrEP assistance program to help with costs associated with clinical services (eg, office visits, laboratory tests) for uninsured and underinsured patients, and there is a large network in New York City of lesbian, gay, bisexual, transgender, and queer (or questioning) (LGBTQ)-affirming and -competent medical providers who prescribe PrEP.

### Development of the E-PrEP Intervention and Attention-Matched Control Conditions

#### Intervention Development Overview

We designed E-PrEP based on formative work conducted by our team [51], in which peers used multiple social media platforms to promote HIV testing using creative messaging and led other social media–based HIV interventions [52,53]. Community-based participatory research methods [47-49,54] guided the development of E-PrEP, with input from a group of 6 YBLGBM intervention development partners experienced in HIV and PrEP outreach in New York City, whom we recruited through an existing HIV outreach initiative for YBLGBM. We selected Facebook and Instagram as the platforms for the intervention, as these are the 2 general social media sites most frequently used by the target population. We did not restrict the intervention to a single platform to provide flexibility, as not all YBLGBM use both sites equally. We developed all contents for both E-PrEP and the control condition (E-Health), and created a content posting and activity guide for all 6 weeks (see Multimedia Appendix 1 for additional details). We standardized E-PrEP in its mode of delivery, types of digital media and contents, and sequence of topics posted and discussed, but during the intervention each peer leader tailored the exact language of each post based on their individual communication style. All materials, including assessments, were mobile phone optimized.

#### Selection of Targets for E-PrEP

We selected potentially modifiable targets to inform message content based on the DOI and IMB models. We also incorporated findings from a qualitative study of local YBLGBM [55], a systematic review of barriers to and facilitators of PrEP, which included a systematic content analysis of online posts about PrEP by men who have sex with men [56], and input from our intervention development partners. Messages were presented using digital media (eg, text, pictures, infographics, and video clips) and posted online based on findings from prior Web-based interventions[57,58] and on social media marketing principles [59]. We designed the content to engage participants in online discussions about HIV prevention, PrEP, and health care [57]. To retain YBLGBM and prevent intervention fatigue [7,60], peer leaders and participants were encouraged to post other items of interest, regardless of their relevancy to HIV or PrEP (eg, pop-culture posts, pictures from recent events, or discussion of current news) [10,61].

#### Development of E-PrEP Intervention Contents

We used an iterative and participatory approach to inform the contents of each E-PrEP online post. First, the research team and our intervention development partners created a digital media library of PrEP educational contents by searching social media (Facebook, Instagram, Twitter, and YouTube) and websites with publicly posted and shareable information about PrEP and accessing health care in the United States. Next, we elicited feedback from the intervention development partners about the following attributes for each media item: their overall thoughts, the comprehensibility, aesthetic appeal, engagability, and informativeness of the item, whether the intervention development partners would actually share the media item, and whether they thought their YBLGBM friends would be likely to view or click on the media item. Then, we took the highest-rated items and mapped them onto a matrix including DOI and IMB domains, as well as barriers to and facilitators of PrEP, to ensure that all relevant topics were covered.

#### Specific E-PrEP Components

Table 1 [62-70] lists examples of E-PrEP intervention contents. The content, formats, and mode and timing of delivery of E-PrEP were informed by the intervention development partners during the intervention development phase, with ongoing input from the peer leaders during intervention implementation. Existing local resources, LGBTQ-friendly clinics, and an existing LGBTQ patient navigator in New York City were highlighted as part of the online content to ensure that people knew how to access care if desired. We only listed clinical resources that accepted new patients, accepted Medicaid or uninsured patients, and were already prescribing PrEP.

#### Development of the Attention-Matched Control Condition

E-Health focused on a broad range of health topics prioritized by the peer leaders assigned to this arm, but did not include any contents about HIV or PrEP. The peer leaders randomly assigned to the E-Health control condition were informed at the first meeting that they would be creating a 6-week social media campaign focusing on health issues they viewed as a priority for YBLGBM within their communities. They chose to cover the following topics: depression, anxiety, suicide, intimate partner violence, drug use, social acceptance, and awareness of sexually transmitted infections (excluding HIV). We designed the E-Health timeline to match the E-PrEP intervention timeline for both time and day of posts and frequency of posts. Similarly to the development of E-PrEP, peer leaders compiled publicly available digital media contents addressing the selected health topics, and then as a group finalized materials to be posted during the online campaign. As with E-PrEP, standardized E-Health contents were posted by peer leaders, framed using their own words. At the end of the trial, peer leaders and participants randomly assigned to E-Health were exposed to all E-PrEP contents.

#### Setup of Web-Based Intervention Sites

We established E-PrEP (intervention) and E-Health (control) private online communities (either a private Facebook group or a private Instagram feed) for each peer leader. We also formed 2 separate private Facebook groups (1 for each condition) for peer leaders, led by a peer facilitator. In these groups, peer leaders could share additional materials, troubleshoot potential issues, and communicate with other peer leaders, the peer facilitator, and a research assistant assigned to that condition. We used third-party content management software (Buffer [71]) to facilitate content posting so that all posts could be prescheduled by peer leaders for the 6-week intervention duration and published at the same day and time in both conditions.

**Table 1:** Empowering with PrEP^a^ (E-PrEP) weekly topics, theoretical domains, and barriers or facilitators targeted.

Week	Weekly theme	DOI^b^ stage	DOI or IMB^c^ domains [62]	Potentially modifiable barrier or facilitator targeted	Example of messaging or contents posted by peer leaders
1	PrEP awareness	Knowledge, Interest	Perceived attributes, Sociocultural norms (DOI); Information, Motivation (IMB)	Lack of PrEP knowledge Low perceived risk	“The government wouldn’t want half the world to contract HIV [emoji face with rolling eyes]. What other myths have you heard about PrEP?” [63] “If you’re dtf let’s talk about it #getprepped #lets talk about it #nycgay” [64] (peers facilitate ongoing discussions on ways to protect, including PrEP)
2	How to talk about sex and PrEP	Knowledge	Perceived attributes, Sociocultural factors (DOI); Information, Behavioral skills (IMB)	Do not know how to get PrEP	“There are some things to consider when taking PrEP, but there are people to answer your questions What questions do you have? #askyourdoctor #getprepped” [65]
3	Talking to partners and friends	Interest	Sociocultural factors (DOI); Motivation (IMB)	Perceived stigma of using PrEP	“Having a positive partner could be the new norm. What makes it hard to bring up PrEP with your partners? #preplove #hivlove #grindrlovestory #getprepped” (clip from video with serodiscordant couple) [66]
4	Overcoming barriers to PrEP	Interest	Perceived attributes (DOI); Information, Motivation (IMB)	Potential side effects	“If you take it at night, how will you feel side effects? Get protected while you sleep #getprepped, What other tips do you have to avoid side effects?” [67]
5	How to get on PrEP?	Knowledge, Decision	Information, Behavioral skills (IMB)	Ability to navigate health care system	“What information should you have handy before you call to make a doctor’s appointment?? Your home address, phone number, date of birth, and insurance information. You’ll be asked why you are making the appointment. They just need the basics, like ‘I want to make an appointment to get on PrEP to prevent HIV.’ Tell the scheduling person if you’re only available certain days or times.” [68]
6	Finding a doctor to prescribe you PrEP and affording PrEP	Decision, Implementation	Perceived attributes (DOI); Behavioral skills (IMB)	Accessing PrEP- or LGBTQ^d^-friendly provider Cost	“DM us your zip code if you want to get on PrEP, and we’ll send you a list of docs in your area! Or Follow this link to find PrEP providers in your county.” [69] “In New York most people can get PrEP for free or cheap, regardless of your insurance status! If you have insurance, including Medicaid, your PrEP will likely be covered. If not, we can help you figure out your options, even if you’re uninsured! Call/text Eric at xxx if you have questions.” [70]

^a^PrEP: preexposure prophylaxis.

^b^DOI: diffusion of innovation model.

^c^IMB: information-motivation-behavioral skills model.

^d^LGBTQ: lesbian, gay, bisexual, transgender, and queer (or questioning).

#### Pretesting and Refining of E-PrEP

To pretest and refine E-PrEP, the intervention development partners each recruited 2 YBLGBM participants from their networks. These participants completed an online consent process and baseline survey, and then received an additional link to join an unlisted private Facebook group where all E-PrEP contents were posted over a 6-week period. The pretest participants provided feedback about contents, process, and acceptability, and also provided suggestions for improvement to all aspects of the intervention through three ways: (1) ongoing feedback elicited by the intervention development partners on the posted E-PrEP contents using open-ended questions in the Facebook group, (2) a brief online acceptability and usability survey at the end of the 6-week period, and (3) an in-person focus group with the intervention development partners and 8 of the pretest participants. Pretest participants received a US $25 debit card as an incentive for their participation. Based on feedback from pretest participants, we refined the E-PrEP intervention by modifying post contents (eg, replacing contents that elicited negative reactions or were considered stigmatizing).

### Implementation of the Intervention

#### Peer Leader Recruitment and Randomization

We recruited 10 YBLGBM peer leaders through advertisements via emails to local youth and LGBTQ-focused community organizations, word-of-mouth through key informants in local YBLGBM communities, and targeted advertisements on Facebook and Instagram. All advertisements directed potential peer leaders to a brief online screening survey. Inclusion criteria for peer leaders were (1) having more than 500 online friends or followers on Facebook or Instagram, (2) using either Facebook or Instagram daily, (3) having positive attitudes about PrEP, (4) residing in New York City, (5) identifying as black or Latino, (6) being sexually active with men in the past year, (7) being fluent in English, (8) being between 18 and 34 years of age, (9) being willing to and feeling comfortable posting and discussing health issues (including sexual health and HIV) with friends on Facebook or Instagram, (10) being able to commit to meeting weekly for 12 weeks for training and intervention implementation, and (11) being able to provide consent. A study coordinator telephoned individuals meeting eligibility to provide further information about the study (eg, that this was a research study and they were study participants as well) and to assess interest and availability. After recruiting peer leaders over a 5-week period, we randomly assigned them to the 2 arms. Peer leaders were blinded to their study condition and were informed that their participation was to help refine and launch an online health promotion campaign for YBLGBM in their online social networks. Each arm was facilitated by a peer facilitator (who also identified as a black or Latinx GBM and was experienced in group facilitation for HIV prevention with YBLGBM in New York City) and supported by a research staff member.

#### Training and Finalizing Intervention Contents for E-PrEP (Intervention Arm)

**Figure 2:**
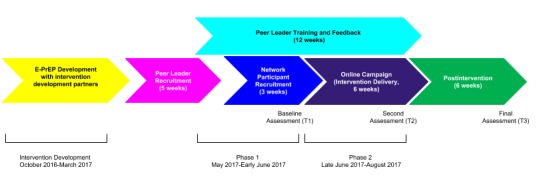
Timeline for intervention development and trial implementation. E-PrEP: Empowering with PrEP; T: time.

We used a participatory process to refine and implement the study. Peer leaders met weekly over a 12-week period. Meetings took place for 3 hours in the evenings at a Wi-Fi–equipped community health center that was easily accessible by public transportation. As Figure 2 displays, the first 6 weeks were dedicated to training and intervention refinement activities (phase 1) and the second 6 weeks were the active intervention delivery period (phase 2).

At the first training session (phase 1), peer leaders randomly assigned to E-PrEP were informed that the overall goal of the campaign was to help prevent HIV in our communities by disseminating accurate information about PrEP to their networks and help link individuals to primary care or PrEP care through an online outreach campaign. During phase 1, peer leaders received training in online recruitment, HIV prevention outreach, PrEP, social media–based outreach and engagement, Buffer software (for scheduling the online posts), and research ethics. As part of the training and intervention refinement, peer leaders reviewed the previously selected and prepiloted E-PrEP digital media materials and made changes or additions as they deemed necessary. During the intervention period (phase 2), peer leaders posted content and provided ongoing feedback to the investigators (see Intervention Procedures, below). In addition to eliciting ongoing feedback during the intervention period (phase 2), we obtained feedback on peer leaders’ experiences participating in the study after study completion using a structured Web-based survey and a semistructured focus group discussion. We collected this information to guide future online outreach practices and capture any relevant issues that emerged during the intervention period but was not captured in our weekly discussions.

#### Training in Research Ethics for Peer Leaders

Peer leaders went through a community research ethics training adapted from a World Health Organization training for lay community members [72]. The training covered topics in the history of research ethics, confidentiality, vulnerable participants, ethical recruitment practices, protecting against risks, minimizing risks, and personal safety and conduct. This training had previously been used with community partners for whom the Collaborative Institutional Training Initiative course [73] was inaccessible due to literacy or language barriers. We also had ongoing discussions throughout the project period in online privacy and security issues. Peer leaders were informed that if there were any clinical questions or questions they did not know the answers to posted, they should send a message with a copy of the question to their peer facilitator and research staff associated with their group, who would obtain the answers to the questions.

#### Compensation for Peer Leader Participants

Peer leaders were considered to be research participants and were compensated for their time and incentivized to actively participate, by receiving increasing amounts of incentive for each week they attended the weekly in-person meetings (up to US $485 via a debit card) over the course of phases 1 and 2. Peer leaders could participate in makeup sessions if needed by meeting one-on-one with the research staff member assigned to their arm. Because peer leaders prescheduled all posts using the Buffer scheduling software, intervention delivery did not have to rely on peer leaders conducting core intervention activities (ie, daily posts in the private online group) outside of the weekly meetings. Peer leaders were encouraged, though, to foster discussions on each post by posing questions to their groups or sharing their thoughts and eliciting feedback from group members.

#### Network Study Participants, Recruitment, and Randomization

Peer leaders, after completing training, recruited network study participants via their existing online social networks to complete an online eligibility screener and baseline survey, using individualized links (Figure 2). Potential network study participants were directed to a Web-based informed consent form, followed by a screener that continued seamlessly into the baseline survey (for eligible network study participants) (using Qualtrics survey software) or an exit page indicating ineligibility. After completion of the baseline survey, network study participants were directed to join the private online intervention group of the peer leader who had recruited them (either a Facebook private group or a private Instagram feed as selected by the peer leader) and were considered to be enrolled after joining the group. Network study participants received an incentive of US $20 after joining the online group.

Inclusion criteria for network study participants were (1) identifying as or assigned male at birth, (2) self-identifying as black or Latino, (3) being 18 to 29 years of age, (4) being fluent in English, (5) being HIV uninfected or unknown by self-report, (6) residing in New York City, (7) having a Facebook or Instagram account, (8) having had insertive or receptive anal sex with a male partner in the past 12 months, and (9) having had 1 of the following in the past 12 months: condomless anal intercourse, anal sex with more than 3 men, bacterial sexually transmitted infection diagnosis (syphilis, gonorrhea, or chlamydia), or a sex partner who was at least 10 years older [74].

We used a cluster-randomized design [34] for several reasons. As we are testing a peer-based social network intervention, keeping study participants clustered with the peer leaders who recruited them maintains ecologic validity and approximates real-world circumstances. This approach also helps minimize contamination within peer networks and is consistent with the DOI model, which highlights social connections in the diffusion process [41,62]. The main drawback to this design is intracluster correlation, such that we cannot assume independence among participants within peer groups. To address this, we will conduct a series of sensitivity analyses (see Analytic Plan, below).

#### Fraudulent or Duplicate Responses

To address potentially fraudulent or duplicate responses, we excluded individuals from the same internet protocol address, recognizing that this approach may inadvertently exclude individuals who simply were sharing a Wi-Fi network. Additionally, we asked for Facebook and Instagram usernames to verify participant identity [75-80]. A research assistant reviewed and approved all requests for entry to the private groups by (1) verifying that the Facebook or Instagram account was already connected to the peer leader who recruited them, (2) asking participants to respond to a private direct message from the research assistant, and (3) insuring that participants had more than 50 friends or followers. We used this last criterion to avoid potentially fraudulent participants who may have developed a new social media account just for the intervention and would thus be unlikely to regularly log in and be exposed to contents being published during the intervention period.

#### Intervention Procedures

After completing recruitment (over a 3-week period), YBLGBM peer leaders launched the intervention by posting materials according to the timeline developed during the training and intervention refinement period (Multimedia Appendix 1). We held weekly project meetings with peer leaders assigned to both E-PrEP and E-Health to ensure appropriate implementation and to discuss logistic, ethical, or other issues that may have arisen. Each group met on different days of the week with different research staff members to help minimize potential contamination. Content for both arms was posted over a 6-week period using Buffer [71], which allowed all the posts to be prescheduled and published in the private groups using the peer leaders’ existing accounts. This approach ensured standardized publication of contents (ie, at the same time and day for each post) and reduced reliance on peer leaders being asked to post contents every day. We collected outcome data from the network study participants via online surveys at baseline, 6 weeks, and 12 weeks. Participants were given US $20, $30, and $40 online gift cards as incentives after completion of assessments at baseline, 6 weeks, and 12 weeks, respectively.

### Measures

#### Online Surveys

We administered online surveys to collect self-reported data at the 3 time points (baseline, 6 weeks, and 12 weeks). We collected data on PrEP use and intention to use, PrEP knowledge and attitudes, self-efficacy for PrEP care, sexual behaviors, sexually transmitted infection and HIV testing, and other covariates listed in Table 2. At each assessment period, participants were sent automated email reminders, followed by direct social media or text message reminders (sent by study staff 24 hours later if surveys were not completed). We repeated these reminders every 2 days until assessments were completed, for a period of 2 weeks. To assess possible contamination between study arms, the 6-week assessment displayed random samples of campaign posts from both E-PrEP and control arms to all participants and assessed recall [34,81,82].

#### Online Engagement Metrics

We collected number of posts viewed, comments, and likes for each participant at 6 weeks. For each post in each of the private peer Facebook groups, we manually collected view data in a spreadsheet by documenting whether a participant had viewed the post or not, coded as 0 (not viewed) and 1 (viewed). For participants using Instagram, we used the same approach to document likes and comments for each of the posts. We also extracted additional engagement data (comments, likes, and reactions) using Grytics software (1339 SAS) for the Facebook groups (not available for Instagram). Grytics is a third-party analytics app that extracts Facebook group engagement data. At the time of the intervention, Grytics did not extract group members’ profile data such as age or gender. Sharing of posts on Facebook was disabled for the trial to reduce potential contamination.

#### Outcomes

Our primary outcomes are (1) self-reported PrEP uptake or intention (measured by indicating either current use of PrEP or intention to use PrEP in the next month) [83], and (2) change on the PrEP motivational cascade [84] at 12 weeks. Secondary outcomes are PrEP knowledge [85], PrEP-related stigma [86], attitudes about PrEP, and access to primary or sexual health care [87]. We will also explore potential changes in social network factors (eg, social support) and whether social media engagement correlated with the primary and secondary outcomes.

### Analytic Plan

#### Feasibility

To test feasibility, we will assess online process measures, including participation rate (number of individuals screening eligible and then ultimately joining the study) and retention metrics (number of participants actively leaving or “unjoining” a social media study site and number of respondents completing follow-up assessments).

#### Acceptability

We will evaluate acceptability by assessing engagement activity (eg, number of posts viewed, number of individuals commenting or liking posts), satisfaction with the intervention, and willingness to continue participating if it were an option, joining a similar study again, and likelihood of recommending friends to join this study if it were an option.

#### Primary and Secondary Outcomes Analysis

First, we will compare groups for equivalence at baseline, using chi-square test, *t* tests, or nonparametric tests as appropriate. In addition, we will determine whether subgroups (eg, grouped by social media platform used, peer leader group, race/ethnicity, and sexual orientation) differ with respect to the primary and secondary outcomes. Relevant differences will be used as covariates in subsequent models. To compare differences in outcomes between arms over time, we will use repeated-measures mixed-effects logistic models, with the treatment arm as a fixed effect, peer leaders as a random intercept, repeated measures from a same participant as another random intercept, and time (as a linear term) as an independent variable. We will also look at residual plots to determine whether we need to include nonlinear terms for time.

**Table 2:** Survey domains for the Empowering with PrEP^a^ (E-PrEP) cluster-randomized controlled trial.

Measures	Week		
	0	6	12
Sociodemographic information	Yes	No	No
Social media access and use	Yes	Yes	Yes
PrEP knowledge, attitudes, and self-efficacy	Yes	Yes	Yes
Stigma (HIV, PrEP, and sexuality related)	Yes	Yes	Yes
Social network factors (social support, PrEP use among friends or partners, relationship with peer leader)	Yes	Yes	Yes
Health care access and use	Yes	Yes	Yes
Sexual health (partners, condom use, sexually transmitted infection history, and HIV testing)	Yes	Yes	Yes
Mental health, alcohol, and substance use	Yes	Yes	Yes
Contamination measures	No	Yes	Yes
Intervention satisfaction	No	Yes	Yes

^a^PrEP: preexposure prophylaxis.

Next, we will use hierarchical models to examine the role of secondary outcomes as potential mediators of change over time; for example, are there differences by condition in PrEP knowledge or attitudes that account for differences in the outcomes? Finally, we will assess the impact of the exposure arm (E-PrEP or E-Health control) and selected covariates (significant in the bivariate analysis at *P*<.15) on the primary and secondary outcomes using mixed-effects models.

#### Online Engagement

To assess the impact of online process and engagement metrics on the outcomes, we will assess in the E-PrEP arm whether intervention exposure and engagement metrics correlate with the primary outcomes of uptake of PrEP and intention to use PrEP. In exploratory analyses, we will assess whether group activity (ie, total number of network participant reactions and posts) correlate with the primary outcomes. To overcome possible limitations due to intracluster correlations, we will also include a modified sensitivity analysis that examines outcomes in relation to participants’ network size and perceived affinity to the peer leaders.

### Challenges and Limitations

#### Attrition

Unlike in prior internet-based interventions, we proposed to use online venues already frequented by YBLGBM and to push intervention components to private home pages, visible only to participants, thus obviating the need to return repeatedly to specific study sites and potentially facilitating engagement. Peer leaders were familiar to participants and served as both recruiters and messengers, delivering contents framed in the peer leader’s communication style. We believe these design considerations will help mitigate attrition, observed in prior Web-based interventions.

#### Contamination

The internet’s strength is that it delivers multidimensional intervention components [88]. Social media’s strength in diffusing innovations is that it facilitates information sharing within and between networks at a pace not previously possible. The nature of social media means there may likely be contamination between study arms. We minimize this to the extent possible by randomizing by peer leader, blinding peer leaders and study participants, and limiting access to the private study sites and contents. Additionally, peer leaders all agreed to not share any contents of materials being posted in their private Facebook or Instagram study groups until after the 12-week assessment.

## Results

Over a period of 5 weeks, from May to June 2017, we recruited, enrolled, and randomly assigned 10 peer leaders (Table 3). From June to July 2017, over a period of 3 weeks, the 10 peer leaders recruited and enrolled a total of 152 network participants (range 4-33 per peer leader; Table 4). Intervention follow-up was completed in November 2017, with analyses ongoing.

**Table 3:** Baseline characteristics of peer leaders in the Empowering with PrEP^a^ (E-PrEP) study.

Characteristics		E-PrEP group (n=5)	Control group (n=5)
Age (years), mean (SD)		24.6 (6.23)	26.4 (5.74)
**Gender identity, n (%)**			
	Male	5 (100)	4 (80)
	Gender nonbinary or gender queer	0	2 (40)
**Residence, n (%)**			
	Bronx	3 (60)	3 (60)
	Brooklyn	0	1 (20)
	Manhattan	2 (40)	1 (20)
**Race/ethnicity, n (%)**			
	Latinx/Hispanic	3 (60)	4 (80)
	Non-Hispanic black	2 (40)	1 (20)
**Sexual orientation, n (%)**			
	Gay/homosexual	3 (60)	3 (60)
	Queer	2 (40)	1 (20)
	Bisexual	1 (20)	1 (20)
**Education level, n (%)**			
	High school or less	1 (20)	2 (40)
	Some college and higher	4 (80)	3 (60)
**Employment^b^, n (%)**			
	Full-time	1 (20)	1 (20)
	Part-time	3 (60)	0
	Unemployed	0	3 (60)
	Student	2 (40)	1 (20)
No. of Facebook friends, mean (SD)		2532 (1657)	3021(1269)
No. of Instagram followers, mean (SD)		2242 (1455)	1443 (644)
**PrEP status**			
	Ever taken PrEP	1 (20)	0
	Never taken PrEP	4 (80)	5 (100)

^a^PrEP: preexposure prophylaxis.

^b^Results may add up to more than 100%, as participants could choose multiple categories.

**Table 4:** Baseline sociodemographic characteristics of network participants in the Empowering with PrEP^a^ (E-PrEP) study.

Characteristics		E-PrEP group (n=81)	Control group (n=71)
Age (years), mean (SD) *t* test		24.28 (2.8)	23.32 (3.4)
**Gender identity, n (%)**			
	Male	68 (84)	64 (90)
	Female/transfemale	7 (9)	3 (4)
	Transmale	1 (1)	0
	Gender nonconforming or nonbinary	2 (3)	0
	Queer	3 (4)	4 (6)
**Residence, n (%)**			
	Bronx	44 (54)	35 (49)
	Brooklyn	18 (22)	14 (20)
	Manhattan	16 (20)	13 (18)
	Queens	2 (3)	8 (11)
	Staten Island	1 (1)	1 (1)
**Race/ethnicity, n (%)**			
	Latinx/Hispanic	26 (32)	47 (66)
	Non-Hispanic black	55 (68)	24 (34)
**Sexual orientation, n (%)**			
	Gay/homosexual	60 (74)	56 (79)
	Queer	12 (15)	3 (4)
	Bisexual	7 (9)	10 (14)
	Heterosexual/straight	1 (1)	1 (1)
	Other	1 (1)	1 (1)
**Education level, n (%)**			
	High school or less	36 (44)	19 (38)
	Some college	28 (35)	35 (49)
	College and higher	17 (21)	9 (13)
**Income (US $), n (%)**			
	None	12 (15)	14 (20)
	<10,000	26 (32)	15 (21)
	10,000-19,999	11 (16)	10 (14)
	20,000-29,999	13 (16)	6 (8)
	30,000-39,999	11 (14)	16 (23)
	≥40,000	8 (10)	10 (14)
**Employment^b^, n (%)**			
	Full-time	24 (30)	29 (41)
	Part-time	17 (21)	15 (21)
	Unemployed	31 (38)	15 (21)
	Disabled	3 (4)	2 (3)
	Student	12 (15)	13 (18)
**Living situation, n (%)**			
	Don’t have a place to live	4 (5)	2 (3)
	Temporary living situation	15 (19)	7 (10)
	Parents or family	29 (36)	36 (51)
	Partner, boyfriend, or husband	2 (3)	7 (10)
	Roommates	20 (25)	14 (19.7%)
	Alone	11 (14)	4 (5.6%)
	Female partner, girlfriend, or wife	0	1 (1.4%)
**Health insurance, n (%)**			
	Yes	61 (75)	59 (83.1%)
	No	18 (22)	10 (14.1%)
	Don’t know	2 (3)	2 (2.8%)
**Type of health insurance, n (%)**			
	Medicaid	33/61 (54)	29/59 (49)
	Your employer or someone else’s employer	18/61 (30)	18/59 (31)
	Medicare	6/61 (10)	2/59 (3)
	Some other source	2/61 (3)	6/59 (10)
	Don’t know or not sure	2/61 (3)	0

^a^PrEP: preexposure prophylaxis.

^b^Results may add up to more than 100%, as participants could choose multiple categories.

## Discussion

Existing behavioral interventions have had limited success in reducing HIV infections in YBLGBM. The promise of PrEP to reduce HIV transmission will be realized in YBLGBM only if uptake, high adherence, and continued engagement in PrEP care is achieved. A social media–based approach to facilitate PrEP uptake may efficiently identify and reach YBLGBM at high risk and may therefore enhance PrEP adoption by helping foster education, motivation, and skills, and linking individuals to sites where they can receive PrEP. Additionally, the use of peer leaders can help influence PrEP uptake by overcoming barriers to engagement and changing social norms and attitudes [89]. Behavioral interventions using peer leaders have been associated with fostering HIV preventive behaviors [90] and in increased viral suppression in HIV-infected individuals [91]. Thus, an intervention model such as E-PrEP, which leverages both peers and social media, could be rapidly scaled up to help accelerate PrEP uptake.

Rather than being simply another medium for adaptation and implementation of existing interventions designed for in-person contact, social media may be a true game changer [7,9,10,81,88] to engage hard-to-reach individuals. While many studies of Web-based behavioral interventions exist, including some that use social media [53], this is, to our knowledge, one of the first to use social media and peer leaders to facilitate uptake of a biomedical intervention. This study is among the first to design and implement a theoretically grounded and completely Web-based intervention codeveloped by peer leaders to accelerate PrEP uptake. Given the paucity of data regarding social media–based interventions to change health behaviors, specifically about a biomedical HIV prevention tool, E-PrEP highlights an important behavioral intervention strategy for existing and future biobehavioral innovations.

Social media offers the power of scale and efficiency for a large potential impact, even with relatively low-intensity interventions [10,81,92]. Similarly, PrEP, if widely adopted in populations at high risk of HIV, could markedly decrease HIV infection rates. Social media–based, peer-led approaches such as E-PrEP could be used to enhance efforts by community-based and other organizations that use internet-assisted or peer outreach strategies to improve health [9]. Findings from this study may help elucidate diffusion processes and factors affecting PrEP adoption, and will lead to the development of a refined social media–based, peer-led intervention, which can then be tested in a fully powered trial. The insights gained from this study may help produce meaningful interventions for YBLGBM, as well as needed data regarding the application of social media- and technology-based interventions to facilitate health behavior change.

## Appendix

Multimedia Appendix 1 Empowering with PrEP (E-PrEP) 6-week online content posting guide.

Multimedia Appendix 2 Review from funding agency.

## Abbreviations

DOI: diffusion of innovation
E-Health: attention-matched general health control condition
E-PrEP: Empowering with PrEP
GBM: gay, bisexual, and other men who have sex with men
IMB: information-motivation-behavioral skills
LGBTQ: lesbian, gay, bisexual, transgender, and queer (or questioning)
PrEP: preexposure prophylaxis
YBLGBM: young black and Latinx gay, bisexual, and other men who have sex with men
