# Supplementary material for: Empowering With PrEP (E-PrEP), a Peer-Led Social Media–Based Intervention to Facilitate HIV Preexposure Prophylaxis Adoption Among Young Black and Latinx Gay and Bisexual Men: Protocol for a Cluster Randomized Controlled Trial
Source: JMIR Res Protoc. 2018 Aug 28;7(8):e11375. doi: 10.2196/11375 (PMC6134229; doi:10.2196/11375)
Supplement: Multimedia Appendix 1 [file resprot_v7i8e11375_app1.pdf]

	Day 1	Day 2	Day 3	Day 4	Day 5	Day 6	Day 7
**Week 1**	Are you PrEPpared for the unknown? #getprepped #thebluepill 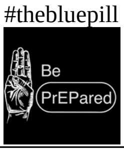	If you're dtf let's talk about it #getprepped #lets talk about it #nycgay 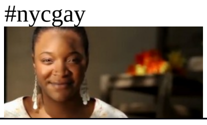	PrEP prevents HIV and uncertainty #getprepped 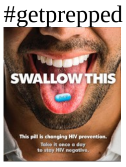	Swallow this: (link to video) 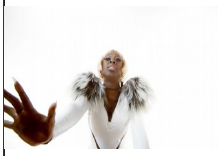		The government wouldn't want half the world to contract HIV 🙄 What other myths have you heard about PrEP? 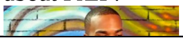	
**Week 2**	Tips for talking to your doctor about PrEP: 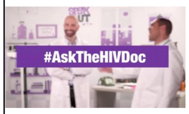	There are some things to consider when taking PrEP but there are people to answer your questions What questions do you have? #askyourdoctor #getprepped 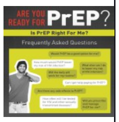		Here's 5 steps to have an open conversation with your doctor about your health issues: 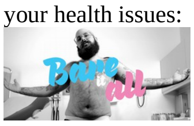		How does this photo make you feel? Do you relate to it? #getprepped #trans # 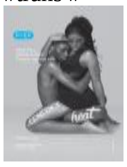	
**Week 3**		PrEP and hormones are like mac n cheese, you can keep on stirring #getprepped #hrt #theT 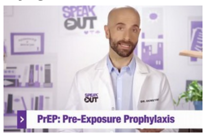		Having a positive partner could be the new norm now with prep. What 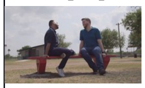 makes it hard to bring up prep with your partners? #preplove #hivlove #grindrlovestory #getprepped		Have you ever had a conversation you're your partners about prep? http://www.hivplusmag.com/prevention/2016/1/29/how-talk-your-partner-about-going-prep	
**Week 4**	Do you think people might have negative reactions to learning you're on prep. Dust off your shoulder. #getprepped #knowyourstatus 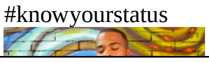		6 things to think about when going on prep: http://www.hivequal.org/hiv-equal-online/6-things-to-think-about-when-considering-prep?slide=1		Side effects might happen, but they can be managed. 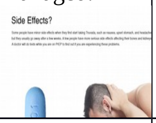		If you take it at night, how will you feel side effects? Get protected while you sleep (ZZZ 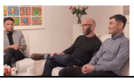
**Week 5**		4 steps to PrEP:<br>1. Find your doctor<br>2. Keep appointments<br>3. Check lab results<br>4. Take your meds		Did you know that NYC Department of Health can answer your questions about PrEP? Text getprep to 69866 for personalized advice on getting prep. [g/#resources]()		So you want to make a doctor's appointment! What information should you have handy before you call? 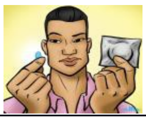	
**Week 6**	Need help finding a doctor who is gets you and can give you prep if you want it? https://www.greaterthan.org/get-prep/	Not everyone feels comfortable telling their regular doctor everything. You can 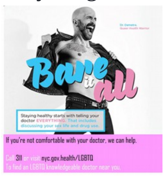 see a different doctor just for your sexual health.	DM us your zip code if you want to get on PrEP, and we'll send you a list of docs in your area! https://www.health.ny.gov/diseases/aids/general/prep/docs/directory.pdf) 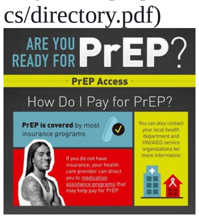	4 Tips to help you Talk to your Doctor about PrEP:<br>1. Be clear.<br>2. Be forthcoming<br>3. Ask questions<br>4. Take notes.<br>For more info, click in the links below:<br>https://buff.ly/2w7TOP9	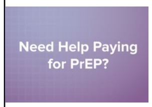 In New York most people can get PrEP for free or cheap, regardless of your insurance status! If not, we can help you figure out your options, even if you're	One in three healthcare providers has not even heard about PrEP, which means you could encounter some resistance if your clinician doesn't have all of the facts.<br>Keep coming back, all day today we will provide you with new facts!!"	Be on the lookout for the second survey ($30!) – If you don't receive the link, let us know! 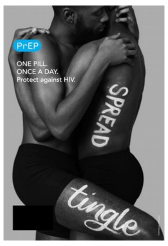
