# Supplementary material for: Empowering With PrEP (E-PrEP), a Peer-Led Social Media–Based Intervention to Facilitate HIV Preexposure Prophylaxis Adoption Among Young Black and Latinx Gay and Bisexual Men: Protocol for a Cluster Randomized Controlled Trial
Source: JMIR Res Protoc. 2018 Aug 28;7(8):e11375. doi: 10.2196/11375 (PMC6134229; doi:10.2196/11375)
Supplement: Multimedia Appendix 2 [file resprot_v7i8e11375_app2.pdf]

1K23MH106387-01 Patel, Viraj

RESUME AND SUMMARY OF DISCUSSION: This excellent candidate is a physician scientist who seeks to develop as an independent investigator with expertise in the development of social media based interventions. In this regard, he has retained an outstanding cast of mentors whose strong letters of support are testimony to their commitment to the success of the candidate. He has presented a very well designed training plan that will impart the skills he seeks. He proposes to investigate factors that are associated with interest in and adoption of pre-exposure prophylaxis (PrEP) uptake among young men of color who have sex with men (YMCSM). He will, then, develop a social media-based peer-led intervention (E-PrEP) to increase PrEP uptake in that population and, finally, he will test the feasibility and preliminary efficacy of E-PrEP for increasing intention to use and adoption of PrEP by YMCSM. The focus of the candidate's research plan is significant in its focus on a population at higher risk of HIV infections and transmission than other groups. Identifying effective prevention methods for this group has remained a challenge; PI has shown some productivity and is evidently dedicated to accomplishing his career goals.

DESCRIPTION: Young men of color who have sex with men (YMCSM, ages 13-29) experience the highest rates of new HIV infections in the U.S., with existing prevention interventions failing to reach most of this hard to reach population. Recent developments in HIV prevention, however, hold promise to help reverse the trend of rising HIV rates in YMCSM, as well as the overall burden of HIV. Pre-exposure prophylaxis (PrEP), an oral antiretroviral regimen taken daily by HIV-uninfected individuals to prevent HIV acquisition, has demonstrated efficacy for reducing HIV acquisition risk in multiple high-risk populations. At the same time, increasingly ubiquitous use of social media (e.g. Facebook, Twitter, online dating sites) by young people, including low- income YMCSM, provides an efficient avenue to identify and reach many otherwise hard to reach YMCSM. The overall goal of this mentored career development award proposal is to develop and pilot-test a social media-based peer-led intervention to promote PrEP uptake in YMCSM. We will use mixed methods and a diffusion of innovation framework to: (1) determine factors associated with interest in and adoption of PrEP among YMCSM; (2) develop Empowering with PrEP (E-PrEP), a social media-based peer-led intervention to increase PrEP uptake in YMCSM; and (3) pilot-test the feasibility and preliminary efficacy of E-PrEP for increasing intention to use and adoption of PrEP by YMCSM. With guidance from a multi-disciplinary team of expert mentors, I will conduct in-person qualitative interviews with users and non-adopters of PrEP to identify current facilitators of and barriers to its uptake. Using the diffusion of innovation theoretical framework to interpret our findings, I will then partner with YMCSM peer leaders to develop a social media-based intervention to facilitate PrEP uptake. The resulting E-PrEP intervention will use online messaging and discussions directed by peer leaders to provide education on PrEP, increase interest in PrEP use, and facilitate access to PrEP. We will then conduct a pilot randomized trial of E-PrEP (versus exposure to online messaging and discussions promoting general health and nutrition) in YMCSM ages 18-29 to assess E-PrEP's feasibility and preliminary efficacy for increasing self-reported intention in and uptake of PrEP among YMCSM. We hypothesize that, compared to a control group exposed to non-overlapping online contents focused on general health topics, participants randomized to E-PrEP will be more likely to express intention to use and to use PrEP. Once we have preliminarily determined an effect size for E-PrEP's efficacy, we will submit an R01 application to test E-PrEP among YMCSM in a multi-center randomized trial.

PUBLIC HEALTH RELEVANCE: Young men who have sex with men of color (YMCSM) in the U.S. experience the highest rates of new HIV infections, with existing prevention interventions failing to reach most of this hard to reach population. Pre- exposure prophylaxis (PrEP), an oral antiretroviral medication taken daily by HIV-uninfected individuals, has shown efficacy in preventing HIV. At the same time, increasingly widespread use of social media (e.g. Facebook, online dating sites) by young people, including YMCSM, provides an efficient avenue to identify and reach large populations of high-risk individuals to rapidly disseminate information about effective HIV prevention tools. This study will use a community based participatory research approach to identify factors impacting PrEP adoption and then design and implement a social media-based peer-led intervention to enhance uptake of this new biomedical innovation for HIV prevention. Study results have the potential to influence PrEP dissemination and help reduce disparities in access to PrEP for hard to reach at-risk populations.

CRITIQUE 1:
Research Plan: 2
Mentor(s), Co-Mentor(s), Consultant(s), Collaborator(s): 1
Environment Commitment to the Candidate: 1

Overall Impact: This is an excellent career award application proposing training and research conducted by a junior, highly promising physician scientist. The training is well structured and should allow the candidate to reach the needed level of technical competency. There are proper provisions for training on the responsible conduct of research with humans. The research component entails a randomized control trial using a Facebook delivered application to increase PrEP uptake in young men of color who have sex with men. The research is significant, innovative, and valid. Overall, the application is very strong and should allow an excellent candidate to progress towards independence.

1. Research Plan:

Strengths

- The application describes the project as the first to try to induce PrEP uptake in this population, particularly using social media. This is significant and innovative.
- The plan is well structured, moving from qualitative community input to develop the program.
- The materials presented with the application seem well developed and indicate that the candidate has taken many important preparatory steps that should ensure success of the project.
- Addressing the needs of the target population in a scientifically valid, innovative way is highly significant.

Weaknesses

- There is a need to specify the techniques/modules of the intervention to ensure they are likely to effectively promote behavior change.

2. Mentor(s), Co-Mentor(s), Consultant(s), Collaborator(s):

Strengths

- The mentoring team is excellent. Dr. Arnsten and Dr. Golub have expertise in medicine and social psychology, respectively. Both are highly accomplished, successful in receiving funding, and very competent mentors.
- There is a supporting team with expertise in social media and data analysis, which should allow the candidate to have adequate mentoring in the different areas concerning the application.

Weaknesses

- None noted

3. Environment and Institutional Commitment to the Candidate:

Strengths

- The environment is excellent, has already supported Dr. Patel in his work.
- The combination of clinical settings and academic units should provide the ideal environment for the training and research components of this application.

Weaknesses

- None noted

Protections for Human Subjects:

Acceptable Risks and Adequate Protections

Data and Safety Monitoring Plan (Applicable for Clinical Trials Only):

Acceptable

Inclusion of Women, Minorities and Children and not IRB Exemption #4.

- Sex/Gender: Distribution justified scientifically
- Race/Ethnicity: Distribution justified scientifically
- Inclusion/Exclusion of Children under 21: Including ages < 21 justified scientifically

Vertebrate Animals:
Not Applicable (No Vertebrate Animals)

Biohazards:
Not Applicable (No Biohazards)

Budget and Period of Support:

Recommend as Requested:

CRITIQUE 2:

Research Plan: 3
Mentor(s), Co-Mentor(s), Consultant(s), Collaborator(s): 1
Environment Commitment to the Candidate: 1

Overall Impact: The candidate seeks to become an independent clinical investigator with expertise in social media based interventions that are developed through CBPR processes. The candidate has great depth of experience across fields - medicine, CBPR methods, community organizing – and demonstrates a high degree of commitment, although his publication record is limited. The mentor team and career development plans are also very strong and highly conducive to the candidate's success. The research plan is clear and highly innovative. This candidate should be expected to contribute much to the field.

1. Research Plan:

Strengths

- Young minority MSM are a highly significant population in need of HIV prevention services.
- The investigator's prior research with social media interventions is a clear strength of the study.
- The steps in the research plan are well thought out.
- The use of diffusion of innovations theory is innovative in this context, and has demonstrated efficacy in other non-PrEP strategies.

Weaknesses

- The selection of intentions to use PrEP is not ideal for providing support to a large R01 trial. It is unclear which barriers the investigator believes will prevent MSM who "intend" to use PrEP from actually using it.

2. Mentor(s), Co-Mentor(s), Consultant(s), Collaborator(s):

Strengths

- The candidate has very strong letters of support from experts in all of the necessary fields: clinical, statistics, intervention development, PrEP, HIV risk behavior research, research ethics, and technology based health research.

Weaknesses

- None noted

3. Environment and Institutional Commitment to the Candidate:

Strengths

- The research and training environment at Einstein / Montefiore is superb. The institution has made a strong commitment to the candidate, assuring at least 80% time devoted to research regardless of the decision on this award.

Weaknesses

- None noted.

Protections for Human Subjects:

Acceptable Risks and Adequate Protections

- Offering E-PrEP to completers of the control arm is a strength.

Data and Safety Monitoring Plan (Applicable for Clinical Trials Only):

Acceptable

- No concerns

Inclusion of Women, Minorities and Children and not IRB Exemption #4.

- Sex/Gender: Distribution justified scientifically
- Race/Ethnicity: Distribution justified scientifically
- Inclusion/Exclusion of Children under 21: Including ages < 21 justified scientifically
- Black and Latino MSM ages 18-29

Vertebrate Animals:

Not Applicable (No Vertebrate Animals)

Biohazards:

Not Applicable (No Biohazards)

Budget and Period of Support:
Recommend as Requested:

CRITIQUE 3:

Research Plan: 1
Mentor(s), Co-Mentor(s), Consultant(s), Collaborator(s): 1
Environment Commitment to the Candidate: 1

Overall Impact: This K-23 (Career Development Award) application from an Assistant Professor of General Internal Medicine aims to enhance the skills needed to become an independent investigator in bio-behavioral HIV interventions for MSM and Transgender populations. The applicant is very well qualified and has demonstrated commitment in bio-behavioral HIV intervention for MSM, and is focused on becoming an independent investigator. The application provides a strong mentorship plan, a schedule of classes and seminars, and a multi-phased developmental and pilot research project grounded in behavioral theory. The specific milestones for evaluation of the candidate's education and skills development are weak.

1. Research Plan:

Strengths

- The research project addresses significant and innovative questions and is guided by an appropriate theoretical model. The study is generally clearly and adequately articulated, and strong.
- The PI will address MSM populations which are at high risk and not well understood.
- The PI is addressing new HIV prevention techniques such as use of PrEP.
- The PI is examining the use of social media as an HIV prevention tool.
- The PI is examining D.O.I. theory
- The PI is learning skills in CBPR

Weaknesses

- None noted

2. Mentor(s), Co-Mentor(s), Consultant(s), Collaborator(s):

Strengths

- The PI proposes mentorship from excellent mentors. The mentors provide letters of strong support for the career development.

Weaknesses

- None noted

3.Environment and Institutional Commitment to the Candidate:

Strengths

- The environment is strong as is the institutional commitment to the applicant

Weaknesses

- None noted

— A **Protections for Human Subjects:**

Acceptable Risks and Adequate Protections

Data and Safety Monitoring Plan (Applicable for Clinical Trials Only):

Not Applicable (No Clinical Trials)

Inclusion of Women, Minorities and Children

G3A - Only Men, Acceptable

M2A - Only Minority, Acceptable

C1A - Children and Adults, Acceptable

- 18-21 included

Vertebrate Animals:

Not Applicable (No Vertebrate Animals)

Biohazards:

Acceptable

Resource Sharing Plans:

Acceptable

Budget and Period of Support:

Recommend as Requested:

THE FOLLOWING RESUME SECTIONS WERE PREPARED BY THE SCIENTIFIC REVIEW OFFICER TO SUMMARIZE THE OUTCOME OF DISCUSSIONS OF THE REVIEW COMMITTEE ON THE FOLLOWING ISSUES:

PROTECTION OF HUMAN SUBJECTS: ACCEPTABLE

INCLUSION OF WOMEN PLAN (G3A): ACCEPTABLE

INCLUSION OF MINORITIES PLAN (M2A): ACCEPTABLE

INCLUSION OF CHILDREN PLAN (C1A): ACCEPTABLE

COMMITTEE BUDGET RECOMMENDATIONS: The budget was recommended as requested.

NIH has modified its policy regarding the receipt of resubmissions (amended applications). See Guide Notice NOT-OD-14-074 at http://grants.nih.gov/grants/guide/notice-files/NOT-OD-14-074.html. The impact/priority score is calculated after discussion of an application by averaging the overall scores (1-9) given by all voting reviewers on the committee and multiplying by 10. The criterion scores are submitted prior to the meeting by the individual reviewers assigned to an application, and are not discussed specifically at the review meeting or calculated into the overall impact score. Some applications also receive a percentile ranking. For details on the review process, see
http://grants.nih.gov/grants/peer_review_process.htm#scoring.

MEETING ROSTER

Behavioral and Social Science Approaches to Preventing HIV/AIDS Study Section
AIDS and Related Research Integrated Review Group
CENTER FOR SCIENTIFIC REVIEW
BSPH
July 07, 2014 - July 08, 2014

CHAIRPERSON

FLYNN, PATRICK M, PHD
PROFESSOR AND DIRECTOR
INSTITUTE OF BEHAVIORAL RESEARCH
DEPARTMENT OF PSYCHOLOGY
TEXAS CHRISTIAN UNIVERSITY
FT WORTH, TX 76109

MEMBERS

ALBARRACIN, DOLORES , PHD
PROFESSOR
ANNENBERG SCHOOL FOR COMMUNICATION
UNIVERSITY OF PENNSYLVANIA
PHILADELPHIA, PA 19104

ALEXANDRE, PIERRE K, PHD *
ASSOCIATE PROFESSOR
DEPARTMENT OF MENTAL HEALTH
BLOOMBERG SCHOOL OF PUBLIC HEALTH
JOHNS HOPKINS UNIVERSITY
BALTIMORE, MD 21205

BANKOLE, AKINRINOLA , PHD
DIRECTOR
INTERNATIONAL RESEARCH
THE GUTTMACHER INSTITUTE
NEW YORK, NY 10005

BLANKENSHIP, KIM M, PHD
PROFESSOR AND CHAIR
DEPARTMENT OF SOCIOLOGY
AMERICAN UNIVERSITY
WASHINGTON, DC 20016

BOEKELOO, BRADLEY O, PHD *
PROFESSOR
DEPARTMENT OF BEHAVIORAL AND COMMUNITY
HEALTH
SCHOOL OF PUBLIC HEALTH
UNIVERSITY OF MARYLAND
COLLEGE PARK, MD 20742

BROWN, LARRY K, MD *
PROFESSOR
DEPARTMENT OF PSYCHIATRY AND HUMAN BEHAVIOR
SCHOOL OF MEDICINE
BROWN UNIVERSITY
PROVIDENCE, RI 02912

CHERNER, MARIANA , PHD *
ASSOCIATE PROFESSOR
DEPARTMENT OF PSYCHIATRY
UNIVERSITY OF CALIFORNIA, SAN DIEGO
SAN DIEGO, CA 92103

COLLINS, REBECCA L, PHD *
DIRECTOR
HEALTH PROMOTION AND DISEASE
PREVENTION PROGRAM
RAND
SANTA MONICA, CA 90407

DICKSON-GOMEZ, JULIA B, PHD *
PROFESSOR
DEPARTMENT OF PSYCHIATRY AND BEHAVIORAL
MEDICINE
CENTER FOR AIDS INTERVENTION RESEARCH
MEDICAL COLLEGE OF WISCONSIN
MILWAUKEE, WI 53202

FEASTER, DANIEL J, PHD
ASSOCIATE PROFESSOR
DIVISION OF BIOSTATISTICS
DEPARTMENT OF PUBLIC HEALTH SCIENCES
UNIVERSITY OF MIAMI
MIAMI, FL 33136

GALEA, SANDRO , MD, DRPH *
GELMAN PROFESSOR AND CHAIR
DEPARTMENT OF EPIDEMIOLOGY
MAILMAN SCHOOL OF PUBLIC HEALTH
COLUMBIA UNIVERSITY
NEW YORK, NY 10032

HALLFORS, DENISE DION, PHD *
SENIOR RESEARCH SCIENTIST
MATERNAL AND CHILD HEALTH
PACIFIC INSTITUTE FOR RESEARCH AND EVALUATION
CHAPEL HILL, NC 27514

HARAWA, NINA THAWATA, PHD
ASSOCIATE PROFESSOR
CENTER FOR HIV IDENTIFICATION, PREVENTION,
AND TREATMENT SERVICES
CHARLES R DREW UNIVERSITY OF MEDICINE AND
SCIENCES
LOS ANGELES, CA 90059

HAVENS, JENNIFER R, PHD
ASSOCIATE PROFESSOR
DEPARTMENT OF BEHAVIORAL SCIENCE
COLLEGE OF MEDICINE
UNIVERSITY OF KENTUCKY
LEXINGTON, KY 40504

KALICHMAN, SETH C, PHD *
PROFESSOR
DEPARTMENT OF PSYCHOLOGY
UNIVERSITY OF CONNECTICUT
STORRS, CT 06269

KOBLIN, BERYL A, PHD
LABORATORY HEAD
LABORATORY OF INFECTIOUS DISEASE PREVENTION
THE NEW YORK BLOOD CENTER
NEW YORK, NY 10021

KURTZ, STEVEN P, PHD
PROFESSOR AND DIRECTOR
CENTER FOR APPLIED RESEARCH ON SUBSTANCE USE
AND HEALTH DISPARITIES
NOVA SOUTHEASTERN UNIVERSITY
CORAL GABLES, FL 33134

LAI, SHENGHAN , MD *
PROFESSOR
DEPARTMENT OF PATHOLOGY
SCHOOL OF MEDICINE
JOHNS HOPKINS UNIVERSITY
BALTIMORE, MD 21287

LEVY, JUDITH A, PHD *
DIRECTOR
FOGARTY AIDS INTERNATIONAL
TRAINING AND RESEARCH PROGRAM
UNIVERSITY OF ILLINOIS AT CHICAGO
CHICAGO, IL 60612

LI, LI , PHD
PROFESSOR
DEPARTMENT OF PSYCHIATRY
AND BEHAVIORAL SCIENCES
UNIVERSITY OF CALIFORNIA, LOS ANGELES
LOS ANGELES, CA 90024

LOUE, SANA , PHD, JD *
PROFESSOR
DEPARTMENT OF EPIDEMIOLOGY AND BIOSTATISTICS
CENTER FOR MINORITY HEALTH
SCHOOL OF MEDICINE
CASE WESTERN RESERVE UNIVERSITY
CLEVELAND, OH 44106

MACQUEEN, KATHLEEN M, PHD
SENIOR SOCIAL SCIENTIST
BEHAVIORAL AND SOCIAL SCIENCES
FAMILY HEALTH INTERNATIONAL
DURHAM, NC 27713

MARSHAL, MICHAEL P, PHD *
ASSOCIATE PROFESSOR
DEPARTMENT OF PSYCHIATRY
SCHOOL OF MEDICINE
UNIVERSITY OF PITTSBURG
PITTSBURGH, PA 15213

MOBLEY, LEE RIVERS, PHD *
ASSOCIATE PROFESSOR
SCHOOL OF PUBLIC HEALTH
GEORGIA STATE UNIVERSITY
ATLANTA , GA 30303

MORRIS, WANDA MARTINA, PHD *
PROFESSOR
DEPARTMENTS OF SOCIOLOGY
AND STATISTICS
UNIVERSITY OF WASHINGTON
SEATTLE, WA 98195

O'CLEIRIGH, CONALL MICHAEL, PHD *
STAFF PSYCHOLOGIST
BEHAVIORAL MEDICINE SERVICE
DEPARTMENT OF PSYCHIATRY
MASSACHUSETTS GENERAL HOSPITAL
BOSTON, MA 02115

PAGE, JOHN BRYAN, PHD *
PROFESSOR AND CHAIR
DEPARTMENT OF PSYCHIATRY AND BEHAVIORAL
SCIENCES
UNIVERSITY OF MIAMI
MIAMI, FL 33124

ROHSENOW, DAMARIS J, PHD *
PROFESSOR
DEPARTMENT OF BEHAVORIAL AND SOCIAL SCIENCES
CENTER FOR ALCOHOL AND ADDICTION STUDIES
BROWN UNIVERSITY
PROVIDENCE, RI 02912

ROTHENBERG, RICHARD B, MD *
PROFESSOR
DEPARTMENT OF FAMILY AND PREVENTIVE MEDICINE
SCHOOL OF MEDICINE
EMORY UNIVERSITY
ATLANTA, GA 30303

SIMONI, JANE MARIE, PHD
PROFESSOR
DEPARTMENT OF PSYCHOLOGY
UNIVERSITY OF WASHINGTON
SEATTLE, WA 98195

STEWART, KATHARINE E, MPH, PHD
ASSOCIATE PROFESSOR AND ASSOCIATE DEAN
DEPARTMENT OF HEALTH BEHAVIOR
AND HEALTH EDUCATION
COLLEGE OF PUBLIC HEALTH
UNIVERSITY OF ARKANSAS FOR MEDICAL SCIENCES
LITTLE ROCK, AR 72205

SWEAT, MICHAEL D, PHD *
PROFESSOR
DEPARTMENT OF PSYCHIATRY
AND BEHAVIORAL SCIENCES
MEDICAL UNIVERSITY OF SOUTH CAROLINA
CHARLESTON, SC 29426

TESTA, MARIA L, PHD *
SENIOR RESEARCH SCIENTIST
RESEARCH INSTITUTE ON ADDICTIONS
UNIVERSITY AT BUFFALO
THE STATE UNIVERSITY OF NEW YORK
1021 MAIN STREET
BUFFALO, NY 14203

VALENTE, THOMAS W, PHD *
ASSOCIATE PROFESSOR
DEPARTMENT OF PREVENTIVE MEDICINE
KECK SCHOOL OF MEDICINE
UNIVERSITY OF SOUTHERN CALIFORNIA
LOS ANGELES, CA 900349045

VASSILEVA, JASMIN L, PHD *
ASSISTANT PROFESSOR
DEPARTMENT OF PSYCHIATRY
COLLEGE OF MEDICINE
UNIVERSITY OF ILLINOIS, CHICAGO
CHICAGO, IL 60612

ZULE, WILLIAM A, DRPH *
SENIOR HEALTH ANALYST
DEPARTMENT OF HEALTH, SOCIAL ECONOMIC
RESEARCH
RESEARCH TRIANGLE INSTITUTE INTERNATIONAL
RESEARCH TRIANGLE PARK, NC 27709

SCIENTIFIC REVIEW ADMINISTRATOR

GUERRIER, JOSE H, PHD
SCIENTIFIC REVIEW OFFICER
CENTER FOR SCIENTIFIC REVIEW
NATIONAL INSTITUTES OF HEALTH
BETHESDA, MD 20892

GRANTS TECHNICAL ASSISTANT

LAFONTANT, MARIE
EXTRAMURAL SUPPORT ASSISTANT
CENTER FOR SCIENTIFIC REVIEW
NATIONAL INSTITUTES OF HEALTH
BETHESDA, MD 20892

* Temporary Member. For grant applications, temporary members may participate in the entire meeting or may review only selected applications as needed.

Consultants are required to absent themselves from the room during the review of any application if their presence would constitute or appear to constitute a conflict of interest.
